# A Virtual, Group-Based Expressive Writing Intervention for Survivors of Adolescent and Young Adult Cancer: Protocol for a Single-Arm Feasibility Study

**DOI:** 10.2196/93460

**Published:** 2026-06-12

**Authors:** Eunju Choi, Michael E Roth, Eileen Hacker, Yisheng Li, Qian Lu

**Affiliations:** 1 Department of Nursing, The University of Texas MD Anderson Cancer Center, 1515 Holcombe Blvd., Houston, TX, 77030, United States, 1 832-794-2033; 2 Department of Health Disparities Research, The University of Texas MD Anderson Cancer Center, Houston, TX, United States; 3 Division of Pediatrics, The University of Texas MD Anderson Cancer Center, Houston, TX, United States; 4 Department of Biostatistics, The University of Texas MD Anderson Cancer Center, Houston, TX, United States

**Keywords:** adolescent and young adult, cancer survivors, expressive writing, feasibility study, acceptability, quality of life, peer support, virtual intervention, group-based intervention, psychosocial intervention

## Abstract

**Background:**

Survivors of adolescent and young adult (AYA) cancer (ages 15‐39 y at diagnosis) face persistent psychosocial challenges, including isolation from peers, identity disruption, and unmet supportive care needs. Approximately 90,000 AYAs are diagnosed with cancer annually in the United States, and 86% survive at least 5 years. However, few age-appropriate, scalable, and developmentally tailored online psychosocial interventions exist for this population. Expressive writing (EW) shows promise for improving psychological well-being in survivors of AYA cancer, but traditional individual approaches do not address the preference of AYAs for peer support. A virtual, group-based EW intervention combining private emotional processing with facilitated peer discussion may address multiple unmet needs.

**Objective:**

The primary objective is to evaluate the feasibility and acceptability of a virtual, group-based EW intervention tailored for survivors of AYA cancer. Secondary objectives are to explore preliminary changes in quality of life and psychosocial outcomes, including perceived stress, coping self-efficacy, cancer-related intrusive thoughts, self-compassion, and posttraumatic growth.

**Methods:**

This single-arm feasibility study enrolls 30 survivors of AYA cancer who were diagnosed at ages 15 to 39 years; are 18 to 39 years old at study entry; are 1 to 5 years postdiagnosis; can speak, read, and write in English; and have regular internet access. Participants are assigned to 1 of 3 groups of 10 on the basis of developmental-stage preferences: emerging adults (18‐25 y), young adults (26‐39 y), or a mixed-age group (18‐39 y). The 8-week intervention consists of 4 private writing sessions of 20 minutes each (odd-numbered weeks), alternating with 4 facilitated group discussions on a closed Facebook platform (even-numbered weeks). All procedures are conducted online, with questionnaires and private writings delivered via REDCap (Research Electronic Data Capture). Primary outcomes include feasibility (success criteria: ≥75% of participants complete assessments; ≥75% complete ≥1 private writing sessions; ≥70% complete all private writing sessions; ≥75% complete ≥1 group discussions; ≥70% complete all group discussions) and acceptability (success criterion: mean score ≥3 on a 0‐4 scale). Secondary and exploratory outcomes are assessed at baseline and at 1-month and 3-month follow-ups. Qualitative data are collected through semistructured interviews, open-ended responses, private essays, and group posts or comments and analyzed using the constant comparative method.

**Results:**

This study was funded in September 2024. Recruitment was completed between June 25 and July 7, 2025. Participants are currently completing 3-month follow-up assessments. Data management is expected to be completed by May 2026, and the results will be submitted for publication by the end of 2026.

**Conclusions:**

This study provides critical feasibility and acceptability data for the first developmentally adapted, virtual, group-based EW intervention for survivors of AYA cancer. The findings will inform the optimization and design of a randomized controlled trial to evaluate the intervention’s efficacy in improving outcomes for this population.

## Introduction

### Background and Rationale

Survivors of adolescent and young adult (AYA) cancer (ages 15‐39 y at diagnosis) face distinct psychosocial challenges that persist long after treatment completion. In this protocol, “survivorship” refers to the period beginning at diagnosis and extending through and beyond active treatment [1]. Approximately 90,000 AYAs are diagnosed with cancer annually in the United States [2], and 86% survive at least 5 years [2], resulting in over 2 million survivors of AYA cancer currently living in the United States [3]. During the transition from active treatment to survivorship, AYAs often experience identity disruption and developmental delays [4-6], social isolation from peers [4,6,7], substantial unmet needs for psychosocial support [6,8], and limited access to age-appropriate support services [5,9,10]. These challenges significantly impair quality of life (QOL) [11,12].

The National Cancer Institute (NCI) defines AYAs as individuals aged 15 to 39 years [13], spanning 3 distinct developmental stages: adolescence (15‐17 y, characterized by parental dependence and peer influence), emerging adulthood (18‐25 y, characterized by exploration and transition to independence), and young adulthood (26‐39 y, characterized by established relationships and career development) [14]. Despite these fundamental differences, most research has not stratified outcomes or tailored interventions by developmental stage.

To be most effective, psychosocial interventions for survivors of AYA cancer should be tailored to their preferences. AYAs value convenience and virtual delivery of interventions [15,16], and 95% express willingness to participate in internet-based interventions [17]. As “digital natives” who spend substantial time on social media [18], AYAs also prefer group-based interventions that provide peer support, which is one of their most frequently reported unmet needs [6,19]. A growing body of evidence supports the use of digital psychosocial interventions for cancer survivors broadly. Recent systematic reviews and meta-analyses have demonstrated that digital health interventions, including web-based platforms, mobile applications, and videoconference-delivered programs, can improve QOL, reduce anxiety and depression, and decrease psychological distress in adult cancer populations [20,21]. However, this evidence base is derived predominantly from older adult cancer populations and may not generalize to AYAs, who differ in developmental stage, psychosocial needs, and digital engagement patterns [22].

Among the few digital interventions developed specifically for AYA cancer survivors, peer support has emerged as a promising approach, with recent trials of group online peer support [23], peer mentoring [24], and online group-based cognitive behavioral therapy with peer discussion [25,26] demonstrating preliminary benefits. One psychosocial intervention that can be delivered via the internet and has been studied in cancer survivors is expressive writing (EW). EW, in which individuals are asked to write about their deepest thoughts and feelings [27], has produced improvements in physical and psychological symptoms among older cancer survivors [28]. Two studies showed that EW decreased distress while increasing QOL and coping abilities in AYAs [29,30]. Our team conducted a feasibility study of individual-based EW among 30 AYA cancer survivors who completed weekly writing sessions for 3 weeks. Results showed improvement in QOL (Cohen *d*=0.73) at the 1-month follow-up, with high acceptability [31]. However, in the classic EW paradigm, in which EW is a private exercise focused on disclosing one’s deepest thoughts and feelings about traumatic experiences, a social support component is lacking. Consistent with this limitation, AYAs in our feasibility study expressed a desire to connect with other AYA survivors and share experiences. However, no existing intervention has integrated private EW with facilitated group peer discussion in a single framework. Together, these findings and the broader literature highlighting peer support as a critical unmet need among AYAs motivated the development of the current virtual, group-based intervention that integrates private EW with facilitated peer discussion and has the potential to better align with AYA developmental needs and preferences.

### Conceptual Framework

**Figure 1.**
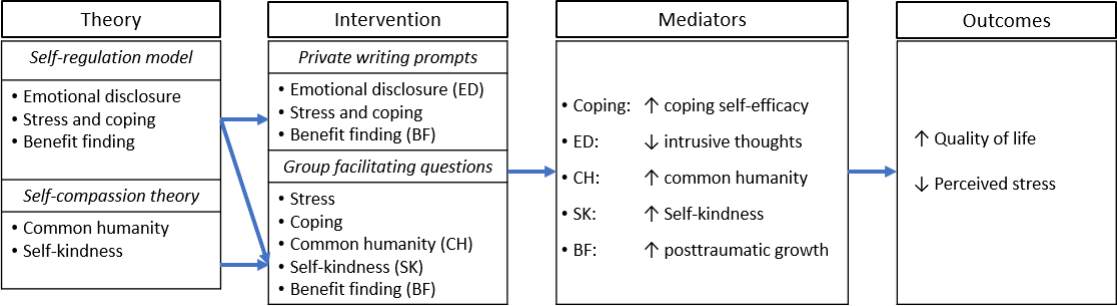
Conceptual framework for group-based expressive writing intervention.

The intervention design is guided by 2 integrated theoretical frameworks (Figure 1). The private writing component is guided by the self-regulation model [32], which posits that actively inhibiting thoughts and feelings about stressful experiences requires physiological effort that accumulates over time, contributing to chronic stress and adverse health outcomes. Writing about these experiences reduces the cognitive burden of inhibition and facilitates cognitive reappraisal, defined as the process of positively reframing stressors to derive meaning and insight [33]. In the EW part of the intervention, the sequence of writing prompts progresses from emotional disclosure to coping exploration to benefit finding and integration.

The group discussion component is guided by self-compassion theory [34], which emphasizes 2 constructs particularly relevant to AYA cancer survivors: common humanity (recognizing shared struggles) and self-kindness (compassionate self-regard). AYA cancer survivors frequently report feeling isolated from same-age peers and disconnected from their precancer identities [4,6], making these constructs particularly relevant to this population. The facilitated peer discussion questions are designed to promote common humanity by encouraging participants to recognize shared experiences, foster self-kindness through supportive and compassionate reflections, and encourage reciprocal peer support while addressing AYA-specific psychosocial challenges.

By combining private self-regulatory writing with facilitated peer discussion designed to cultivate common humanity and self-kindness, this intervention targets both intrapersonal meaning-making and interpersonal support needs. These theoretical foundations directly inform the study objectives: to evaluate whether this integrated approach is feasible and acceptable to AYA survivors (primary aim) and to explore preliminary changes in QOL and psychosocial outcomes consistent with the theoretical frameworks, including perceived stress, coping self-efficacy, intrusive thoughts, self-compassion, and posttraumatic growth (secondary aim). In this paper, we describe the research protocol.

## Methods

This protocol is reported in accordance with the Standard Protocol Items: Recommendations for Interventional Trials (SPIRIT) 2013 guidelines [35]. The completed SPIRIT checklist is provided in Checklist 1.

### Study Design and Setting

This is a single-arm feasibility study without a comparator group and is designed to evaluate the feasibility and acceptability of a virtual, group-based EW intervention for survivors of AYA cancer. The primary objective at this stage is to establish feasibility and acceptability prior to designing a randomized controlled trial. This is an open-label study; no blinding is used, as the single-arm design and behavioral nature of the intervention make blinding of participants or study personnel unnecessary. All procedures are conducted remotely. Surveys and private writing activities are delivered via the secure web-based platform REDCap (Research Electronic Data Capture), and group discussions take place in closed, private Facebook groups.

### Intervention Development

The intervention was developed through an iterative, multistage process. The theoretical foundation drew on the self-regulation model of EW [32] and self-compassion theory [34], as described in the *Introduction*. A targeted literature review identified key features of effective psychosocial interventions for survivors of AYA cancer, including the importance of peer support [6,19], virtual delivery [15,16], and structured writing protocols of sufficient dose and duration [36]. Preliminary research by our team, including a feasibility study of individual-based EW among 30 survivors of AYA cancer [31], provided initial evidence of acceptability and improvement in QOL, while also revealing participants’ desire for peer connection. The iterative development and modification process for the EW intervention is described in detail elsewhere [37].

The initial intervention design was presented to the Young Adult Advisory Council of The University of Texas MD Anderson Cancer Center, which comprised survivors of AYA cancer and providers from the MD Anderson Cancer Center AYA clinic, who provided feedback on acceptability, intervention frequency and duration, writing prompts, and group composition considerations. Specifically, they endorsed the alternating format of private writing followed by group discussion as a way to balance personal reflection with peer interaction. All feedback was systematically reviewed and integrated into the intervention design.

Following this stakeholder engagement, a run-in pilot with 10 survivors of AYA cancer was conducted between January and February 2025 to test logistics and obtain participant feedback. The run-in pilot yielded 2 critical insights that informed the final protocol. First, a group of 5 participants was insufficient to sustain engagement when even 1 or 2 members did not post in a given week. This finding led to the decision to increase the group size to 10 participants, providing a buffer for natural fluctuations in participation while still maintaining a manageable volume of interaction. Second, survivors diagnosed less than 1 year prior struggled with participation due to the demands of intensive treatment and acute adjustment challenges. Time since diagnosis emerged as a more important determinant of feasibility than other clinical factors (such as cancer type, disease status, or treatment status). The eligibility criterion was therefore revised to require that participants be a minimum of 1 year postdiagnosis, reflecting the recognition that different survivorship phases may require different intervention approaches [38,39]. Skill-focused interventions targeting immediate practical concerns may be more appropriate during active treatment [40,41], whereas reflective, meaning-making interventions like this study may be better suited for survivors who are further out from the acute phase of care [42,43]. Additional refinements included improvements to the writing prompt content and facilitated discussion questions based on participant input.

### Participants

Inclusion criteria are age 15 to 39 years at diagnosis; age 18 to 39 years at study entry; 1 to 5 years postdiagnosis (participants may be receiving active treatment, including maintenance therapy, or may be off treatment); ability to speak, read, and write in English; and access to the internet. Exclusion criteria are diagnosis of nonmelanoma skin cancer and any major mental health disorder (eg, schizophrenia or bipolar disorder), as determined by medical records or self-report. Table 1 provides detailed inclusion and exclusion criteria with rationales. The exclusion of individuals with major mental health disorders was implemented for 2 reasons. First, the group-based format of the intervention requires participants to engage in facilitated peer discussion about emotionally sensitive topics, and the presence of acute psychiatric symptoms (eg, psychosis, mania, or active suicidal ideation) could compromise both individual safety and group functioning. Second, the exploratory outcome measures in this pilot study (eg, perceived stress, self-compassion, and posttraumatic growth) could be significantly confounded by concurrent major psychiatric conditions, making it difficult to evaluate preliminary signals of intervention effects. This criterion was determined by clinical judgment at the time of screening rather than by formal diagnostic assessment, consistent with the approach used in similar pilot trials of psychosocial interventions for AYA cancer survivors [23,26].

**Table 1.** Inclusion and exclusion criteria and rationales.

Criterion	Details	Rationale
Inclusion		
Age at diagnosis	15‐39 years	Aligns with National Cancer Institute (NCI) definition of AYAs^a^
Age at study entry	18‐39 years	Participants must be legal adults (≥18 y)
Time since diagnosis	1‐5 years	≥1 year ensures completion of acute treatment and initial adjustment; ≤5 years targets the early survivorship phase
English literacy	Can speak, read, and write in English	Required for current delivery (future iterations will include other languages)
Internet access	Regular access via smartphone, tablet, or computer	Required for virtual intervention; ~97% of US adults aged 18‐39 years have internet access
Exclusion		
Cancer type	Nonmelanoma skin cancer	Has treatment trajectory and psychosocial profile different from those of other cancers
Mental health	Major mental health disorder (eg, schizophrenia or bipolar disorder)	Requires specialized clinical support and could pose safety or feasibility concerns for the intervention

a AYA: adolescent and young adult.

A total sample size of 30 was chosen using a CI approach [44] to provide adequate precision for feasibility metrics. With 30 participants, a 95% CI for a 75% (or 50%) completion rate would have a half-width of approximately 15.5% (or 17.9% in the worst-case scenario), which is acceptable for estimating feasibility [45,46]. This sample size also permits estimation of preliminary effect sizes to inform power calculations for future trials.

### Recruitment and Consent

Participants were recruited from June 25, 2025, to July 7, 2025, via multiple channels. Research data coordinators systematically screened patient records in the MD Anderson AYA clinic via the electronic medical record system to identify potentially eligible survivors. Identified patients were contacted via email, text, or phone. Simultaneously, study flyers were posted in the MD Anderson AYA Survivors Facebook group (with >1800 members), and interested individuals could contact the research team directly. Intensive recruitment minimized the wait time between consent and group start. Once eligibility was confirmed, participants provided informed consent electronically through Epic (Epic Systems Corporation). During the consent process, participants were informed that they would be asked about their group composition preferences (similar-age, mixed-age group, or no preference) but might not be assigned to their preferred group depending on the overall enrollment distribution.

To enhance the diversity of the sample, recruitment materials were distributed through social media channels. Demographic and clinical characteristics of enrolled participants (including age, sex, race or ethnicity, cancer type, and time since diagnosis) were tracked and will be reported to characterize the composition of the sample. However, given the pilot nature and small sample size of this study, purposeful stratification by sociodemographic or clinical subgroups may not be feasible.

### Group Composition

During the enrollment process, participants indicated their preference for being in a group with participants of a developmentally similar-age group (either 18‐25 y or 26‐39 y), a mixed-age group (18‐39 y), or no preference. This preference-based approach was chosen for 2 reasons. First, the developmental heterogeneity within the AYA age range (spanning late adolescence to early midlife) means that survivors at different life stages may face distinct psychosocial concerns (eg, identity formation and educational disruption for younger AYAs versus career, financial, and parenting concerns for older AYAs) [14]. Allowing participants to self-select into age-based or mixed-age groups reflects the person-centered design principles recommended for AYA intervention research [47]. Second, understanding AYA preferences for group composition is itself a relevant question for intervention optimization, as group cohesion and perceived relevance are known to influence engagement in group-based psychosocial interventions [48]. As an exploratory aim, this study will also examine whether preference-based age group assignment is feasible and whether group composition (younger AYA, older AYA, or mixed-age) is associated with differential patterns of engagement and acceptability.

### Intervention

The 8-week intervention consists of four 2-week sessions. Each session includes 1 private EW task (during weeks 1, 3, 5, and 7), followed by 1 facilitated group discussion activity (during weeks 2, 4, 6, and 8). The use of 4 EW tasks is consistent with the dose most commonly used in the EW literature and has been shown to be both feasible and psychologically beneficial in cancer survivors and other healthy populations [36].

Following group assignment, participants receive weekly writing prompts via REDCap, write privately for a minimum of 20 minutes, and submit their essays electronically. This 20-minute duration aligns with the writing duration in the standard EW protocol, which typically ranges from 15 to 30 minutes, and is considered sufficient to promote emotional disclosure, narrative organization, and cognitive reappraisal [49-51]. Private essays are not automatically shared with the group; participants control what (if anything) they choose to share from their writing. The writing prompts are designed to progress through thematic phases: week 1 focuses on emotional disclosure (writing about one’s cancer experiences and their impact), week 3 focuses on coping exploration (how the participant managed challenges during cancer), week 5 focuses on benefit finding (identifying any positive changes or appreciation gained through the experience), and week 7 focuses on integration (reflection and advice to others, synthesizing the participant’s journey). Prompts address AYA-relevant concerns such as identity, education or career disruption, relationships, and future uncertainty. Writing topics for each session are outlined in Table 2.

**Table 2.** The 8-week intervention schedule with private expressive writing (EW) topics and group discussion questions.

Session	Theoretical target	EW topic	Facilitating questions for group discussion
1	Emotional disclosure, initial cognitive reappraisal	Week 1: Your cancer experiences and their impact on you and your life	Week 2: Specific difficult moments; life impacts; emotions as AYA^a^; challenges in expressing experiences
2	Stress and coping, meaning-making	Week 3: How you have handled your cancer experiences and learned from them	Week 4: Expected or unexpected challenges and how handled; coping strategies used; changes in coping over time
3	Benefit finding, posttraumatic growth	Week 5: Appreciation	Week 6: Things appreciated more deeply; moments of gratitude; relationships strengthened; strengths discovered
4	Integration, wisdom-sharing	Week 7: Advice	Week 8: Advice to newly diagnosed; advice wish you’d received; message to past self; balancing life demands; relationship changes

a AYA: adolescent and young adult.

Following each private writing week, participants receive a set of facilitating discussion questions via REDCap, along with a link to their closed Facebook group. These questions closely mirror the theme of the preceding private writing prompt and are intended to promote reflection and group interaction. Participants post their responses in the group, sharing only what they feel comfortable disclosing, and they can react to or comment on other group members’ posts. Participation is asynchronous, allowing individuals to contribute at any time during the discussion week. The facilitating questions are designed to promote common humanity by encouraging participants to recognize shared experiences, encourage self-kindness through supportive and compassionate reflections, and foster reciprocal peer support, all while addressing AYA-specific challenges. The facilitating questions are provided in Table 2.

The principal investigator (PI) monitors all groups on a daily basis for content safety and appropriateness. The PI posts weekly reminders and prompts as needed but does not actively lead discussions in a traditional therapy manner; the discussion is intended to be peer-driven rather than therapist-led.

To promote adherence and engagement, the research team implements several strategies: automated email reminders are sent before each activity; text reminders are sent to participants who have not completed a given task; personal follow-up outreach is conducted if a participant missed consecutive activities; and incremental compensation is provided for each completed component. Total possible compensation is US $110 per participant, consisting of US $10 for the baseline questionnaire, US $10 for each of the 4 private writing tasks, US $10 for each of the 4 group discussions, and US $10 each for the 1-month and 3-month follow-up questionnaires. Compensation is prorated so that participants who withdraw early receive credit for the proportions they completed. All payments are delivered as electronic gift cards. There are no restrictions on concomitant care. Participants may continue receiving medical, psychological, or supportive care services throughout the study.

### Outcome Measures

#### Demographic and Clinical Characteristics

Table 3 provides an overview of all study measures and their assessment time points. Demographic characteristics (eg, age, sex assigned at birth, sexual orientation, gender identity, education level, employment status, income, insurance status, relationship status, and cohabitation status) are collected at screening or baseline. Cancer-related clinical information is self-reported (and supplemented by medical record review with consent) and includes age at cancer diagnosis, cancer type, stage at diagnosis, treatment modalities received, time since diagnosis, and time since treatment completion.

**Table 3.** Constructs, instruments, and assessment time points.

Construct (instrument)	Assessment time points											
	Baseline	Week 1	Week 2	Week 3	Week 4	Week 5	Week 6	Week 7	Week 8	After intervention	1M^a^	3M^b^
Demographics	✓											
Clinical information	✓										✓	✓
Group composition preference	✓											
Quality of life (FACT-G)^c^	✓										✓	✓
Perceived stress (PSS-4)^d^	✓										✓	✓
Coping self-efficacy (CBI)^e^	✓										✓	✓
Intrusive thoughts (impact of event scale—revised, intrusion subscale)	✓										✓	✓
Common humanity (self-compassion scale, common humanity subscale)	✓										✓	✓
Self-kindness (self-compassion scale, self-kindness subscale)	✓										✓	✓
Posttraumatic growth (PTGI-9)^f^	✓										✓	✓
Private writing		✓		✓		✓		✓				
Group discussion			✓		✓		✓		✓			
Manipulation check									✓			
Acceptability									✓			
Group cohesion (group cohesiveness scale)									✓			
Interview										✓		

a 1M: 1 month after completion of intervention.

b 3M: 3 months after completion of intervention.

c FACT-G: Functional Assessment of Cancer Therapy-General.

d PSS: Perceived Stress Scale.

e CBI: Cancer Behavior Inventory.

f PTGI: Posttraumatic Growth Inventory.

#### Primary Outcomes

Feasibility is assessed through prespecified quantitative benchmarks. The criteria for success are as follows: at least 75% of participants complete the 1-month and 3-month follow-up assessments; at least 75% of participants complete at least 1 private writing task and ≥70% complete all 4 writing tasks; and at least 75% of participants complete at least 1 group discussion and ≥70% complete all 4 discussions.

Acceptability is assessed through an acceptability questionnaire containing 9 items rated on a 0 to 4 scale [52], with a mean score of 3 or greater indicating an acceptable intervention. In addition, semistructured postintervention interviews explore participants’ experiences with the intervention format, content, and delivery; group composition; perceived changes in themselves; barriers to participation; and suggestions for improvement.

#### Secondary Outcomes

All secondary outcomes are assessed at baseline, 1-month follow-up, and 3-month follow-up using validated instruments.

QOL is measured using the Functional Assessment of Cancer Therapy-General (FACT-G), a 27-item questionnaire that assesses physical, social, emotional, and functional well-being [53]. Each item is rated on a 5-point scale (0=not at all; 4=very much), with higher FACT-G scores indicating better QOL. The FACT-G has been used in diverse samples [53], including AYAs with cancer [31], and has shown satisfactory internal reliability (Cronbach α=0.90) [53].

Perceived stress is measured using a 4-item short form of the Perceived Stress Scale (PSS-4) [54]. Participants report how often they experienced certain feelings and thoughts in the past week on a 5-point scale (0=never; 4=very often), with higher scores indicating greater perceived stress. The PSS-4 has demonstrated adequate internal consistency (Cronbach α range: 0.74‐0.91) in cancer survivor samples [55].

#### Exploratory Mediating Outcomes

Several psychosocial constructs that might mediate intervention effects are also measured at baseline, 1-month, and 3-month follow-ups with validated instruments.

Coping self-efficacy is measured using the Cancer Behavior Inventory (CBI), which contains 12 items assessing confidence in performing behaviors relevant to coping with cancer and its treatment [56]. Each item is rated on a 9-point Likert-type scale from 1 (“not at all confident”) to 9 (“totally confident”). Higher total scores indicate greater coping self-efficacy. The CBI has demonstrated good internal consistency in cancer populations (Cronbach α=0.84‐0.89) [56].

Cancer-related intrusive thoughts are measured using the intrusion subscale of the impact of event scale—revised, focusing on unwanted thoughts related to the cancer experience [57]. This subscale contains 4 items rated on a 5-point scale from 0 (“not at all”) to 4 (“extremely”). Higher scores indicate more frequent or distressing intrusive thoughts; the subscale has shown excellent internal reliability (Cronbach α=0.91) in cancer survivors [57].

Common humanity and self-kindness are measured using the subscales of the self-compassion scale: the common humanity subscale (4 items measuring recognition of shared human experience) and the self-kindness subscale (5 items measuring kindness toward oneself) [58]. Items are rated on a 5-point scale (1=almost never; 5=almost always). Higher subscale scores indicate greater common humanity and self-kindness, respectively. These subscales have demonstrated good internal reliability (Cronbach α=0.80‐0.84) [58].

Posttraumatic growth is measured using the 9-item version of the Posttraumatic Growth Inventory (PTGI-9), which assesses positive changes experienced as a result of facing a traumatic event (in this case, cancer) [59]. Each item is rated on a 6-point scale from 0 (“did not experience this change”) to 5 (“experienced this change to a very great degree”), with higher scores reflecting greater perceived growth. The PTGI-9 has shown high internal reliability (Cronbach α=0.93) [59].

#### Manipulation Check and Group Cohesion

After the final writing task (week 8), participants completed a brief manipulation check consisting of 7 items (each rated 0‐10) evaluating whether the writing exercises were meaningful, personal, and emotional [60]. Participant engagement with the group aspect was assessed using the group cohesiveness scale [61], which measures the participant’s perceived sense of bonding and connection within the group.

#### Qualitative Data

Qualitative data are collected from multiple sources: postintervention one-on-one interviews; open-ended survey responses (eg, feedback questions in the follow-up surveys); the content of participants’ private writing essays; and the posts and comments from the group discussions. These qualitative data provide contextual insights into feasibility, acceptability, and potential mechanisms.

### Data Management and Confidentiality

All study data are collected and managed using REDCap electronic data capture tools hosted on secure, HIPAA (Health Insurance Portability and Accountability Act)-compliant servers at MD Anderson. Participants are assigned unique study ID codes; all survey and quantitative data are identified only by these codes. Personal identifying information (eg, contact information and consent forms) is stored separately in encrypted files accessible only to authorized study personnel. Private writing essays and content from the Facebook group discussions are downloaded and stored on secure, encrypted institutional servers. These text data are also identified by participant code and treated as confidential research data. Only the research team has access to the raw data, and all analyses and reports present aggregated or deidentified information.

### Safety Monitoring

This is a minimal-risk behavioral intervention study. The primary anticipated risk is the possibility of emotional distress from writing about or discussing difficult personal experiences. Several mitigation strategies are in place: participants were informed during the consent process that they should only write or share what they feel comfortable with and that they could skip any activity or question that causes discomfort. The PI monitors the online group daily for any concerning content (eg, indications of severe distress or intent to self-harm). When necessary, the study team will contact participants who express distress and provide information about the MD Anderson AYA clinic social work and counseling services, as well as 24-hour crisis hotline numbers and other mental health resources for additional support. The PI remains available throughout the study period to respond to any participant concerns. If a participant reports significant emotional distress or a clinical concern arises, the PI will assess whether continued participation is appropriate and may recommend that the participant withdraw, with referral to support services. Participants may discontinue the intervention at any time without penalty; outcome data collected prior to withdrawal will be retained for analysis unless the participant requests otherwise. Any adverse events or serious issues are documented and reported to the MD Anderson Institutional Review Board (IRB) according to standard research protocols. Because this is a minimal-risk behavioral intervention with a small sample, a formal data monitoring committee was not required. The PI is responsible for ongoing monitoring of participant safety, and all adverse events are reported to the MD Anderson IRB.

### Statistical Analysis

For quantitative data, feasibility metrics are summarized as proportions with 95% CIs. Descriptive statistics (means, medians, and SDs as appropriate) are used to characterize the sample and engagement patterns. For preliminary outcome exploration, mean scores at each time point (baseline, 1 mo, and 3 mo) are reported for each measure. Within-participant changes from baseline to follow-ups are examined using 2-tailed paired *t* tests for approximately normally distributed variables or Wilcoxon signed-rank tests for nonnormal distributions. Effect sizes (Cohen *d*) with 95% CIs are calculated for key outcome changes to aid in planning future studies. All quantitative analyses are considered exploratory rather than confirmatory. Due to the small sample size of the study, our primary analysis for the secondary outcomes will be based on all observed data, with acknowledgment of the limitations of the approach. If, however, the proportion of missing data is excessively high (eg,≥30%), we may explore multiple imputation approaches to handling the missing data, for example, by assuming a missing-at-random mechanism, as part of our sensitivity analyses. No interim analyses or stopping rules are planned for this pilot feasibility study.

Qualitative data will be collected from 3 sources: (1) participants’ private writing entries, (2) group discussion posts on the online platform, and (3) semistructured exit interviews conducted via Zoom (Zoom Communications, Inc) after the intervention period. These 3 data sources differ in structure and purpose. Private writing entries are individual, unprompted reflections on personal experiences and emotions. Group discussion posts are briefer, naturalistic exchanges generated in real time during the intervention that capture peer interaction and group dynamics. Exit interviews yield in-depth, prompted responses about participants’ overall experiences, perceived benefits, challenges, and recommendations for improvement. Qualitative data will be analyzed using thematic analysis following the 6-phase framework described by Braun and Clarke [62]. Each data source will first be coded separately to preserve its distinct characteristics. Private writings will be analyzed to capture intrapersonal processes such as emotional disclosure, cognitive reappraisal, and meaning-making, consistent with the self-regulation model. Group discussion posts will be analyzed to capture interpersonal processes such as peer support, self-disclosure, and expressions of common humanity and self-kindness, consistent with self-compassion theory. Interview transcripts will be analyzed to capture participants’ reflective accounts of their overall experiences and suggestions for improvement. Two independent coders will code the data, and discrepancies will be resolved through discussion to reach consensus. After initial coding, themes will be compared and integrated across the 3 sources to identify convergent and divergent findings. Themes will be organized around the study’s feasibility and acceptability objectives. To connect qualitative and quantitative findings, a mixed methods integration approach will be used [63]. Qualitative themes related to engagement, satisfaction, and perceived benefit will be examined alongside quantitative feasibility metrics and preliminary outcome data to provide a comprehensive understanding of intervention processes and effects. The semistructured interview guide is provided in Multimedia Appendix 1.

### Ethical Considerations

This study protocol was reviewed and approved by the IRB of The University of Texas MD Anderson Cancer Center (Study ID 2024‐0440). This manuscript reflects protocol version 1.1, dated January 2026. All participants provided electronic informed consent prior to participation. Participants were reminded that their involvement was voluntary and that they could withdraw from the study at any time without penalty. The trial was registered on ClinicalTrials.gov (NCT06434402) before participant recruitment began. Any significant changes to the protocol will be submitted to the MD Anderson IRB for approval and updated on ClinicalTrials.gov. Results will be submitted for publication in a peer-reviewed journal and reported on ClinicalTrials.gov. Participants will receive a summary of the study findings upon completion of the study.

## Results

This study was funded in September 2024. Participant recruitment was completed between June 25, 2025, and July 7, 2025, yielding 30 enrolled participants. All 30 participants were successfully assigned to an intervention group according to the scheme described (2 developmentally stratified groups and 1 mixed-age group). The 8-week intervention phase (private writing and group discussion sessions) was completed by all 3 groups. Follow-up assessments at 3 months after the intervention are currently ongoing. Data processing and management (including quantitative dataset cleaning and qualitative transcription) are expected to be completed by May 2026. The data will be analyzed, and a manuscript on the results and findings is anticipated to be submitted for publication by the end of 2026.

## Discussion

### Principal Considerations

This study protocol describes a novel virtual, group-based EW intervention specifically designed for survivors of AYA cancer. The intervention addresses a critical gap in supportive care for AYAs by combining private, individualized emotional processing with structured peer interaction. In doing so, it targets both cognitive reappraisal and self-compassion mechanisms. By integrating the self-regulation model of EW with self-compassion theory [32,34], the intervention is intended to facilitate not only personal meaning-making but also peer-based social support, which is one of the most frequently reported unmet needs among survivors of AYA cancer [4,6,7].

Several design features of the intervention are tailored to AYA preferences and the developmental diversity within this age range. The fully virtual delivery format provides flexibility and convenience valued by this digitally native population [15,16] while also removing geographic barriers to participation. The asynchronous nature of the online group discussions allows participants to engage at times that fit their schedules, yet it maintains the benefits of peer support and interaction. Moreover, assessing participants’ grouping preferences by developmental stage and attempting to honor these preferences is an innovative approach to promoting group cohesion [17]. AYAs spanning the broad age range of 15 to 39 years can have substantially different life circumstances and concerns [14]; thus, it remains an empirical question whether grouping by closer age range will enhance engagement and outcomes. Future trials may even need to consider additional grouping factors (eg, time since diagnosis or treatment, disease status, cancer type, or key life-stage characteristics) to foster group cohesion and effective peer support. This feasibility study will begin to address that question.

### Limitations

This protocol is a single-arm feasibility study with inherent limitations. Without a control group or randomization, the study cannot determine the efficacy of the intervention; therefore, a randomized controlled trial will be required to test effects on outcomes. With 30 participants, the study is also not powered for definitive hypothesis testing or for comparing subgroups (eg, younger vs older AYAs). There is potential selection bias that may limit generalizability. Participants needed internet access to take part (although 97% of US adults aged 18‐39 y have internet access), and the intervention content was available only in English (future iterations will include translated versions to increase accessibility for non-English speakers). Recruitment was conducted primarily through MD Anderson (augmented by social media outreach), so the sample may not fully represent the broader population of survivors of AYA cancer, especially those without ties to large cancer centers. Demographic and clinical characteristics of enrolled participants will be examined when interpreting feasibility metrics to identify whether additional recruitment strategies are needed for a future trial. Additionally, the 3-month follow-up period is relatively short, providing only preliminary insight into the sustainability of any benefits. Lastly, the exclusion of individuals with major mental health disorders, while appropriate for this pilot stage to ensure participant safety and interpretability of exploratory outcomes, may limit the generalizability of findings. AYA cancer survivors experience elevated rates of depression and anxiety [64,65], and future full-scale trials should consider whether this criterion can be narrowed to exclude only acute safety risks (eg, active suicidal ideation, psychosis) while including individuals with stable, managed mental health conditions such as depression or anxiety. This would improve the representativeness of the sample and the applicability of findings to the broader AYA survivor population.

Despite these limitations, this feasibility study is a crucial step in the development of a new intervention. The study design includes rigorous a priori feasibility and acceptability criteria, validated outcome measures, a mixed methods approach, and stakeholder input at multiple stages. These design elements enhance the quality of the data obtained and will inform meaningful modifications. The knowledge gained will directly guide the optimization and design of a subsequent efficacy trial.

### Conclusions

This feasibility study represents a critical first step in developing a potentially scalable, accessible intervention that addresses the urgent psychosocial needs of survivors of AYA cancer. By grounding the intervention in evidence-based theoretical frameworks, engaging AYA stakeholders throughout the development process, iteratively refining the protocol via pilot feedback, and aligning the format with AYA preferences for virtual, peer-supported engagement, we have laid the groundwork for a novel approach to AYA survivorship care. If feasibility and acceptability are established in this pilot study and preliminary signals of benefit are observed, the findings will directly inform a randomized controlled trial to formally evaluate efficacy. Such a trial could provide much-needed evidence for interventions aimed at improving QOL and reducing psychological distress among the growing population of survivors of AYA cancer. Given the substantial and persistent challenges faced by this group and the current lack of developmentally tailored, evidence-based interventions for this group, this work addresses an important gap in cancer survivorship research and care.

## Supplementary material

10.2196/93460 Multimedia Appendix 1 Poststudy interview guide.

10.2196/93460 Checklist 1 SPIRIT 2025 editable checklist.

## Abbreviations

AYA: adolescent and young adult
CBI: Cancer Behavior Inventory
EW: expressive writing
FACT-G: Functional Assessment of Cancer Therapy-General
HIPAA: Health Insurance Portability and Accountability Act
IRB: Institutional Review Board
NCI: National Cancer Institute
PI: principal investigator
PSS-4: Perceived Stress Scale
QOL: quality of life
SPIRIT: Standard Protocol Items: Recommendations for Interventional Trials

## References

[1] Definitions. National Cancer Institute, Division of Cancer Control and Population Sciences (DCCPS).

[2] (2020). Special section: cancer in adolescents and young adults. https://www.cancer.org/content/dam/cancer-org/research/cancer-facts-and-statistics/annual-cancer-facts-and-figures/2020/special-section-cancer-in-adolescents-and-young-adults-2020.pdf.

[3] Page LL, Devasia TP, Mariotto A, Gallicchio L, Mollica MA, Tonorezos E (2025). Prevalence of cancer survivors diagnosed during adolescence and young adulthood in the United States. J Natl Cancer Inst.

[4] Kim B, White K, Patterson P (2016). Understanding the experiences of adolescents and young adults with cancer: a meta-synthesis. Eur J Oncol Nurs.

[5] Bibby H, White V, Thompson K, Anazodo A (2017). What are the unmet needs and care experiences of adolescents and young adults with cancer? A systematic review. J Adolesc Young Adult Oncol.

[6] Choi E, Becker H, Kim S (2022). Unmet needs in adolescents and young adults with cancer: a mixed-method study using social media. J Pediatr Nurs.

[7] Breuer N, Sender A, Daneck L (2017). How do young adults with cancer perceive social support? A qualitative study. J Psychosoc Oncol.

[8] Zebrack BJ, Corbett V, Embry L (2014). Psychological distress and unsatisfied need for psychosocial support in adolescent and young adult cancer patients during the first year following diagnosis. Psychooncology.

[9] Tsangaris E, Johnson J, Taylor R (2014). Identifying the supportive care needs of adolescent and young adult survivors of cancer: a qualitative analysis and systematic literature review. Support Care Cancer.

[10] Choi E, Becker H, Jung H (2023). Health-related quality of life in adolescents and young adults with and without cancer, using propensity score matching. J Cancer Surviv.

[11] Sawyer SM, McNeil R, McCarthy M (2017). Unmet need for healthcare services in adolescents and young adults with cancer and their parent carers. Support Care Cancer.

[12] Quinn GP, Gonçalves V, Sehovic I, Bowman ML, Reed DR (2015). Quality of life in adolescent and young adult cancer patients: a systematic review of the literature. Patient Relat Outcome Meas.

[13] Cancer stat facts: cancer among adolescents and young adults (AYAs) (ages 15–39). National Cancer Institute.

[14] Arnett JJ (2000). Emerging adulthood. A theory of development from the late teens through the twenties. Am Psychol.

[15] Rabin C, Simpson N, Morrow K, Pinto B (2013). Intervention format and delivery preferences among young adult cancer survivors. Int J Behav Med.

[16] Benedict C, Victorson D, Love B (2018). The audacity of engagement: hearing directly from young adults with cancer on their attitudes and perceptions of cancer survivorship and cancer survivorship research. J Adolesc Young Adult Oncol.

[17] Oswald LB, Victorson DE, Fox RS (2021). Young adult cancer survivors’ preferences for supportive interventions. Psychooncology.

[18] Devine KA, Viola AS, Coups EJ, Wu YP (2018). Digital health interventions for adolescent and young adult cancer survivors. JCO Clin Cancer Inform.

[19] Kent EE, Smith AW, Keegan THM (2013). Talking about cancer and meeting peer survivors: social information needs of adolescents and young adults diagnosed with cancer. J Adolesc Young Adult Oncol.

[20] Zhang Y, Flannery M, Zhang Z (2024). Digital health psychosocial intervention in adult patients with cancer and their families: systematic review and meta-analysis. JMIR Cancer.

[21] Zhang T, Ren Z, Wakefield CE (2025). Are digital psychological interventions for psychological distress and quality of life in cancer patients effective? A systematic review and network meta-analysis. Clin Psychol Rev.

[22] Murphy KM, Siembida E, Lau N, Berkman A, Roth M, Salsman JM (2023). A systematic review of health-related quality of life outcomes in psychosocial intervention trials for adolescent and young adult cancer survivors. Crit Rev Oncol Hematol.

[23] Li L, Duan Y, Cao H (2024). Effect of group online-based peer support intervention on psychological distress of adolescent and young adult cancer patients: a randomized controlled trial. Support Care Cancer.

[24] Brock H, Dwinger S, Bergelt C (2024). Peer2Me—evaluation of a peer supported program for adolescent and young adult (AYA) cancer patients: study protocol of a randomised trial using a comprehensive cohort design. BMC Cancer.

[25] Irestorm E, Wakefield CE, Hetherington K (2026). Recapturing life: virtual peer-based psychological support for adolescent and young adult cancer survivors delivered in the community. J Adolesc Young Adult Oncol.

[26] Sansom-Daly UM, Wakefield CE, Bryant RA (2012). Online group-based cognitive-behavioural therapy for adolescents and young adults after cancer treatment: a multicenter randomised controlled trial of Recapture Life-AYA. BMC Cancer.

[27] Pennebaker JW, Beall SK (1986). Confronting a traumatic event: toward an understanding of inhibition and disease. J Abnorm Psychol.

[28] Henry EA, Schlegel RJ, Talley AE, Molix LA, Bettencourt BA (2010). The feasibility and effectiveness of expressive writing for rural and urban breast cancer survivors. Oncol Nurs Forum.

[29] Peckham JL, Block R, Buchanan M, Pommier S (2017). Unspoken ink: a structured, creative writing workshop for adolescents and young adult cancer patients as a psychosocial intervention. J Adolesc Young Adult Oncol.

[30] Anzeneder S, Secco DE, Mastronuzzi A, Colasanti AR, Gentile S (2018). QOL-35. Expressive writing for adolescents with brain tumor: a case study. Neuro Oncol.

[31] Shin-Cho LJ, Dawkins-Moultin L, Choi E (2025). Feasibility trial of an online expressive writing intervention for young adult cancer survivors. J Adolesc Young Adult Oncol.

[32] Lu Q, Stanton AL (2010). How benefits of expressive writing vary as a function of writing instructions, ethnicity and ambivalence over emotional expression. Psychol Health.

[33] Lazarus RS, Folkman S (1984). Stress, Appraisal, and Coping.

[34] Neff K (2003). Self-compassion: an alternative conceptualization of a healthy attitude toward oneself. Self Identity.

[35] Chan AW, Tetzlaff JM, Altman DG (2013). SPIRIT 2013 statement: defining standard protocol items for clinical trials. Ann Intern Med.

[36] Zachariae R, O’Toole MS (2015). The effect of expressive writing intervention on psychological and physical health outcomes in cancer patients—a systematic review and meta-analysis. Psychooncology.

[37] Choi E, Xu YA, Wong-Meli CC, Roth ME, Li Y, Lu Q (2026). Optimizing feasibility and acceptability of an online expressive writing intervention for survivors of adolescent and young adult cancer: a pilot randomized trial of iterative modifications and outcomes. Psychooncology.

[38] Mullan F (1985). Seasons of survival: reflections of a physician with cancer. N Engl J Med.

[39] Stanton AL (2012). What happens now? Psychosocial care for cancer survivors after medical treatment completion. J Clin Oncol.

[40] Nezu AM, Nezu CM, Felgoise SH, McClure KS, Houts PS (2003). Project Genesis: assessing the efficacy of problem-solving therapy for distressed adult cancer patients. J Consult Clin Psychol.

[41] Antoni MH, Lehman JM, Kilbourn KM (2001). Cognitive-behavioral stress management intervention decreases the prevalence of depression and enhances benefit finding among women under treatment for early-stage breast cancer. Health Psychol.

[42] Marco JH, Llombart P, Romero R (2024). Meaning-centered psychotherapy versus cognitive behavioral therapy for cancer survivors: a randomized controlled trial. Behav Ther.

[43] Stanton AL, Danoff-Burg S, Sworowski LA (2002). Randomized, controlled trial of written emotional expression and benefit finding in breast cancer patients. J Clin Oncol.

[44] Cocks K, Torgerson DJ (2013). Sample size calculations for pilot randomized trials: a confidence interval approach. J Clin Epidemiol.

[45] Hertzog MA (2008). Considerations in determining sample size for pilot studies. Res Nurs Health.

[46] Teresi JA, Yu X, Stewart AL, Hays RD (2022). Guidelines for designing and evaluating feasibility pilot studies. Med Care.

[47] Arora NK, Street RL, Epstein RM, Butow PN (2009). Facilitating patient-centered cancer communication: a road map. Patient Educ Couns.

[48] Burlingame GM, McClendon DT, Yang C (2018). Cohesion in group therapy: a meta-analysis. Psychotherapy (Chic).

[49] Frattaroli J (2006). Experimental disclosure and its moderators: a meta-analysis. Psychol Bull.

[50] Pennebaker JW (1997). Writing about emotional experiences as a therapeutic process. Psychol Sci.

[51] Smyth JM (1998). Written emotional expression: effect sizes, outcome types, and moderating variables. J Consult Clin Psychol.

[52] Collings S, Mathieson F, Dowell A (2012). Acceptability of a guided self-help mental health intervention in general practice. Fam Pract.

[53] Brucker PS, Yost K, Cashy J, Webster K, Cella D (2005). General population and cancer patient norms for the Functional Assessment of Cancer Therapy-General (FACT-G). Eval Health Prof.

[54] Cohen S, Kamarck T, Mermelstein R (1983). A global measure of perceived stress. J Health Soc Behav.

[55] Lee EH (2012). Review of the psychometric evidence of the perceived stress scale. Asian Nurs Res (Korean Soc Nurs Sci).

[56] Merluzzi TV, Nairn RC, Hegde K, Martinez Sanchez MA, Dunn L (2001). Self-efficacy for coping with cancer: revision of the Cancer Behavior Inventory (version 2.0). Psychooncology.

[57] Horowitz M, Wilner N, Alvarez W (1979). Impact of event scale: a measure of subjective stress. Psychosom Med.

[58] Neff KD (2003). The development and validation of a scale to measure self-compassion. Self Identity.

[59] Tedeschi RG, Calhoun LG (1996). The Posttraumatic Growth Inventory: measuring the positive legacy of trauma. J Trauma Stress.

[60] Pennebaker JW, Colder M, Sharp LK (1990). Accelerating the coping process. J Pers Soc Psychol.

[61] Wongpakaran T, Wongpakaran N, Intachote-Sakamoto R, Boripuntakul T (2013). The group cohesiveness scale (GCS) for psychiatric inpatients. Perspect Psychiatr Care.

[62] Braun V, Clarke V (2006). Using thematic analysis in psychology. Qual Res Psychol.

[63] Creswell JW, Clark VLP (2017). Designing and Conducting Mixed Methods Research.

[64] Zhang A, Urban-Wojcik E, Seewald M, Zebrack B (2025). Mental health trajectories among US survivors of adolescent and young adult cancer as they age. JAMA Netw Open.

[65] Osmani V, Hörner L, Klug SJ, Tanaka LF (2023). Prevalence and risk of psychological distress, anxiety and depression in adolescent and young adult (AYA) cancer survivors: a systematic review and meta-analysis. Cancer Med.

