# Supplementary material for: A Virtual, Group-Based Expressive Writing Intervention for Survivors of Adolescent and Young Adult Cancer: Protocol for a Single-Arm Feasibility Study
Source: JMIR Res Protoc. 2026 Jun 12;15:e93460. doi: 10.2196/93460 (PMC13263011; doi:10.2196/93460)
Supplement: Multimedia Appendix 1 [file resprot-v15-e93460-s001.docx]

**Participant Interview Guide**

**Introduction & Verbal Consent**

*“Hello! Thank you for participating in the SHINE AYA Study. We are talking with participants in the SHINE AYA study to get your feedback on the design of the study. Your ideas and thoughts are very important to us and will help us to improve our study. This interview will take about 30-45 minutes, and you will be compensated with a $20 Amazon gift card for your time spent with me. Is now still a good time to chat?”*

IF NO: *“Could we schedule some time next week to chat?”*

IF YES: *“Great! Before we get started, may I record the interview to make sure I don’t miss anything you say? We will only record the audio. “*

[IF YES: TURN ON THE RECORDER]

*“Wonderful. The recorder is on. I want to let you know that everything you tell me will be kept anonymous and confidential. That means that we will never give out your name or connect you to any comments you share with us. After we review the recording and write down what you shared with us, we will delete the recording. We will not include any information about you in the written notes. Please feel comfortable to freely share your thoughts and feelings with us. Also, if you prefer not to answer any question, just let me know, and we’ll skip the question.”*

*“Do you consent to participate in the interview?”*

[IF YES, “*Let’s begin*!”]

[IF NO, ANSWER THEIR QUESTIONS AND CONCERNS.]

*“In this interview, we would like to know your opinions about the content and format of the SHINE AYA study.”*

**Interviewer Note:** Feel free to ask additional follow-up questions as needed. Use this interview guide as a base, not a script you must follow verbatim. As we go through the interview, keep in mind the following goals:

1. Determine if we need to revise the writing prompts or the facilitating questions for group discussion.
2. Identify anything that could better engage and retain participants (i.e., help participants stay in the study and complete all parts).

**Interview Questions**

**Warm-Up:**

*“First of all, thank you for participating in the SHINE AYA Study! Can you share with us ….”*

Q. What your overall experience has been like in this study so far? What made you decide to join the study in the first place?”

[This is just a question to get participant to warm up. If participant starts talking about anything relevant to the questions listed below, please feel free to skip ahead, and circle back to the missed questions later.]

*“Thank you for the feedback. If you don’t mind, I would like to ask some specific questions related to the writing portion of the study.”*

**Private Writing Portion:**

Q. I know it has been a little while since the writing sessions. Can you recall the topics that you were asked to write about during the study?

If Yes 🡪 *“Great! What are some of the writing topics you remember?”*

IF No 🡪 “*That’s okay, it has been a while. Let’s review the materials together. I’m pulling up the prompts on the screen now.” (Share screen and present Week 1 private writing prompt.)*

Q. *[After displaying Week 1 prompt]* How did you feel about this writing prompt? What was it like for you to write about this topic?

[Participants may mention how easy or difficult it was to write, whether they liked the topic, or how it affected their thinking]

Q. *[Present Week 1 group discussion questions for that week.]* How did you feel about Week 1 group discussion questions? What was it like to discuss this topic with others?

Q: What recommendations (if any) do you have for improving the Week 1 writing exercises – both the private writing prompt and the group discussion questions for that week?

[Repeat the above process for **Week 2, Week 3, and Week 4**. For each week, show the private writing prompt and the group discussion questions. Ask how the participant felt about that week’s prompt and discussions, and why. Then ask if they have any suggestions to improve that week’s materials.]

[Stop sharing screen]

**Overall Writing Experience - Private Writing**

*“Now that we have gone through the tasks week by week, I would like to know more about your overall experience with the writings.”*

Q: Overall, how did you feel about the private writing exercises across the four weeks? How helpful or beneficial, if at all, did you find these writing exercises?

*[Possible probe if needed:* “In what ways were they helpful to you? Did they have any impact on your thoughts or feelings outside of the writing sessions?”*]*

Q. Have you continued writing on your own now that the writing portion of the study is over?”

*If YES:* “What kinds of things do you write about now on your own? What topics or issues do you find yourself writing about?”

*If NO:* (Skip to next question.)

Q*.* Were there any other topics that were not covered in these writing sessions that you wish you could have written about? If so, what topics would you have liked to explore?

**Overall Writing Experience - Group Discussion**

Q. How did you feel about having the chance to talk in a group setting about something you had just written privately? What was helpful or not helpful about that experience?”

Q. Did you enjoy participating in the group discussions? What aspects did you like the most, and what aspects were less helpful?”

Q. If we were to run the group discussions again in the future, what changes would you recommend to make them more useful or engaging?”

*[Possible probes if needed:* “Would you change anything about the environment, the platform, the kinds of discussion questions, frequency of interaction, or the way participants were grouped?”]

Q. Looking back on your experience, what role did the group discussions play in your overall study experience? Do you feel they added something unique that private writing alone would not have?

Q. In this study, you received group discussion questions after completing your private writing and shared only what you felt comfortable posting. How would you feel about having the option to share your full private writing directly on the platform? Would that interest you, and why or why not?

**Overall Reflections**

Q. If you had the chance to modify the frequency of the writing tasks, how would you adjust the schedule? Currently it was one private writing and one group discussion every two weeks for eight weeks. How did you feel about that workload? Would you have preferred a different frequency or duration?

Q. If given the choice, would you have liked to receive a copy of your own writings after submitting them each week?

**Group Discussion Portion:**

*“Thank you. Now I’d like to talk about your experience with the group discussion part of the study.”*

Q: Overall, how did you feel about the group discussions on Facebook? What was your experience like participating in the group?”

[Possible probe: “Did you find the group discussions helpful or supportive in any way? Why or why not?”]

Q: Have you ever joined any cancer support group outside of this study (either online or in person)? If so, what has your involvement been like in those groups?

If YES: “How engaged are you in those groups? For example, do you actively post or comment, or do you mostly read what others post (just lurk)?”

If NO: “Okay. (No problem – I was just curious about your outside experiences.)

Q. How did you like using Facebook for the group discussions in this study? What did you like or dislike about using Facebook as the platform?

Q. Are there any other platforms or online communities you would suggest for group discussions and connecting with other participants? For example, some people use Reddit or a dedicated website/app. Do you have any preferences or ideas for a better platform or way to facilitate group interaction?

Q. How did you feel about the role of the facilitator in the group discussions? Did you feel the facilitator was the right level of active or involved, or would you have preferred them to be more or less engaged? What suggestions do you have for improving the facilitator’s role in future groups?

Q. How would you feel about sharing your cancer experiences in a more real-time format—like live group meetings on Zoom or in person—instead of just posting online? What would be your thoughts or concerns about that kind of format?

Q. In the study, you were placed in a group of about 10 participants (including yourself) for the discussions. How did you feel about the size of that group? Did it feel too small, too large, or about right for you?

Q. At the beginning of the study, we asked about your group formation preference regarding age (for example, whether you preferred to be in a group with people of similar age or a mixed-age group). Do you feel that being grouped with survivors of a similar age influenced your group experience or engagement? If so, how? If not, why not?

Q. Beyond age, what other characteristics or elements do you think we should consider when creating participant groups for a study like this? Are there other factors that you believe would make a group more comfortable or supportive?”

*[possible probes:* “For instance, would it be important to you to be grouped with people of the same gender or at a similar life stage (like other college students, or others who have children)? What about grouping by similar cancer experience, such as the same diagnosis, stage, or treatment status?”*]*

Q. Before we move on, is there anything else about the writing tasks or the group discussions that you would like to share or think we should know?

**Feedback on Other Study Aspects**

“Great, thank you. Now I’d like to ask about some other aspects of the study.”

Q. What did you think about the recruitment materials for this study, such as the advertisements or flyer you saw? Did anything in particular catch your attention or could something be improved in how the study was described to you?”

[show the recruitment flyer on the screen as a refresher.]

Q. What were your impressions of the reminder notifications we sent out during the study (for example, reminders to complete your writing or survey)? Were the reminders helpful, annoying, or something in between?”

Do you feel we sent too many, too few, or about the right number of reminders?”

Would you have preferred a different way of being contacted or reminded? For instance, would texts, emails, phone calls, or other methods have worked better for you?

Q. Considering the time you spent on completing the writing tasks and surveys, how do you feel about the compensation you received for participating in the study? Did you find the compensation adequate for the effort and time you put in?

Q. Is there anything else that you would like to share with us about your experience in the study or any suggestions you have for us? This could be absolutely anything that we haven’t covered but you feel is important.

*Q.* We are in the process of forming a Community Advisory Board to help with the next phase of this project. The board will meet occasionally to provide feedback as we revise the study or design the new study. Would you be interested in possibly joining this advisory board?

**Conclusion**

*“We sincerely appreciate your contribution to this study and for taking the time to participate. Please remember that any comments from this conversation used in reports will be kept anonymous. Before we stop the recording, is there anything else you think would be important for us to know?”*

[terminate the recording]

*“Thank you once again for participating and for being so open with your feedback. Before we finish, is there anything you’d like to tell me off the record? (This is just between us and won’t be documented.)”*
